# Anterior cervical discectomy and fusion for the treatment of giant cervical disc herniation

**DOI:** 10.1186/s13018-023-04036-5

**Published:** 2023-09-13

**Authors:** Weibin Liang, Yu Xiong, Yukun Jia, Shaoqiang Li, Kaishuai Zhao, Zhan Peng, Guangye Wang

**Affiliations:** 1 Clinical Medicine School of Shenzhen Bao’an, Guangdong Medical University, Shenzhen, Guangdong Province China; 2 Affiliated Baoan Hospital of Shenzhen, The second school of clinical medicine, Southern Medical University, Shenzhen, Guangdong Province China; 3 Department of Spine Surgery, The People’s Hospital of Baoan Shenzhen, Shenzhen, Guangdong Province China; 4 Department of Rehabilitation, Heshan People’s Hospital, He Shan, Guangdong Province China

**Keywords:** Giant cervical disc herniation, Spine-neck, Surgery, Anterior cervical discectomy and fusion

## Abstract

**Objective:**

Giant cervical disc herniation (GCDH) was defined as a herniated intervertebral disc that accounted for more than 50% of the spinal canal. The purpose of this study was to analyse the feasibility of anterior cervical discectomy and fusion (ACDF) for the treatment of GCDH.

**Methods:**

Patient demographic and imaging data, clinical results, and perioperative complications were analysed retrospectively.

**Results:**

A total of 23 patients were included in the study. Spinal cord recovery pulsation was observed under a microscope in all cases. Postoperative magnetic resonance imaging showed complete decompression of the spinal cord and no residual intervertebral disc. The patients were followed up for 12 to 18 months. The average visual analogue scale score and Neck Disability Index decreased from 8.6 ± 0.5 and 86.0 ± 2.7% to 2.2 ± 0.2 and 26.7 ± 2.0%, respectively, three days after surgery. The average Japanese Orthopedic Association score increased from 6.9 ± 2.1 to 13.9 ± 1.1. The cervical spinal cord function improvement rate was 69.3%. No neurological complications after surgery were observed.

**Conclusion:**

This study shows that ACDF is feasible for the treatment of GCDH disease. The results indicate that this approach can be used to safely remove herniated disc fragments, effectively relieve compression of the spinal cord, and improve neurological function.

**Supplementary Information:**

The online version contains supplementary material available at 10.1186/s13018-023-04036-5.

## Introduction

Giant cervical disc herniation (GCDH) is a relatively rare spinal disease that was first described by Dantas in 1999 [1]. He proposed the classification of cervical disc herniation according to the degree of spinal cord compression and defined GCDH as cervical intervertebral disc herniation accounting for more than 50% of the spinal canal. Compared to small- and medium-sized disc herniation, the neurological defects observed in patients with GCDH are more serious. As the cache space in the spinal canal is relatively narrow, a large volume of herniated disc increases the difficulty of surgical treatment. To the best of our knowledge, to date, there are few studies of the clinical characteristics, treatment, and prognosis of patients with GCDH.

Surgery is the primary treatment for GCDH. However, there remains some controversy regarding the choice of surgical approach and, at present, there is no gold standard approach. Several scholars [2] suggest that posterior laminoplasty should be used to expand the volume of the spinal canal and indirectly relieve spinal cord compression. However, since most herniated intervertebral discs are located in front of the spinal cord, the decompression effect of posterior surgery is poor compared with anterior surgery and, in theory, the maximum distance at which the spinal cord can retreat is about 50% of the sagittal diameter of the spinal canal [3, 4]. When spinal canal stenosis combined with anterior compression invades more than 50% of the spinal canal, even if posterior decompression allows the spinal cord to move backward to the maximum extent, it is still not enough to avoid anterior compression; thus, spinal cord compression remains. Williams et al. [5] removed the nucleus pulposus and other compression directly through anterior cervical corpectomy decompression and fusion (ACCF), but the incidence of complications such as adjacent segmental lesions or pseudoarthrosis was high [6, 7]. Further, compared with anterior cervical discectomy and fusion (ACDF), ACCF sacrifices one extra normal intervertebral disc, which means more fusion segments and a lower range of motion. Recently, several scholars have argued that the combination of the anterior and posterior approach for the treatment of GCDH increases the surgical time, the amount of blood loss during surgery, and the surgical complications.

ACDF is a safe and reliable technique which is considered to be the gold standard for the treatment of single or multi-segmental cervical spondylotic radiculopathy and cervical spondylotic myelopathy [8, 9]. However, to the best of our knowledge, there are few reports on the use of ACDF for the treatment of GCDH.

This study reports the results of 23 patients with GCDH treated with ACDF.

## Material and methods

### Patient data

Each patient’s demographic and clinical characteristics were collected from their electronic medical records. Informed consent was obtained from all patients.

The inclusion criteria were as follows: (1) obvious medullary symptoms and signs; (2) preoperative MRI showing huge intervertebral disc herniation, invading more than 50% of the spinal canal; and (3) no significant improvement in symptoms after routine conservative treatment for more than six months. The exclusion criteria were: (1) vertebral metastatic tumours or primary tumours; (2) cervical skin and soft tissue infection; (3) history of cervical surgery; (4) severe cardiopulmonary dysfunction; or (5) obvious oppression of the nerve root or spinal cord by a posterior osteophyte. A total of 23 patients were enrolled in this study, including 14 males and 9 females aged from 29 to 85 years, with an average age of 51.8 ± 14.8 years. Six patients were Frankel grade C, and 17 patients were Frankel grade D. There were 3 cases of asymptomatic patients with no clear history of trauma; 5 cases of asymptomatic patients with post-traumatic onset of symptoms; 11 cases of mildly symptomatic patients with worsening of symptoms after trauma; 2 cases of mildly symptomatic patients with recent worsening of symptoms but without a clear history of trauma; and 2 cases of chronic course. The patient's symptoms are mainly characterized by spinal cord or nerve root injury as the main manifestation, accompanied by numbness and weakness of the upper limbs, a feeling of girdle in the trunk, and unsteady walking, hyperreflexia of tendons, increased muscle tone, positive pathological signs, etc. The clinical features of the patients are shown in Table 1.

**Table 1:** Clinical characteristics of patients with Giant cervical disc herniation (*n* = 23)

Patient	Age (years)	Sex	Symptoms	Duration of symptoms (months)	Frankel	VAS	NDI	JOA	Level	Follow-up (months)
1	85	F	Neck pain arm dysaesthesia	12	C	4.2	32.6	7	C3/4 C4/5	15
2	41	M	Progressive leg weakness	10	D	7.9	39.9	9	C3/4	12
3	68	F	Neck pain bilateral leg paraesthesia	5	D	7.4	52.9	5	C3/4	12
4	72	F	Neck pain	8	D	6.5	34.0	10	C3/4	13
5	49	F	Neck pain arm dysaesthesia	6	D	9.3	44.9	7	C3/4 C4/5 C5/6	14
6	42	M	Neck pain arm dysaesthesia	11	D	9.3	69.4	10	C3/4 C4/5 C5/6	16
7	56	M	Neck pain, arm and leg dysaesthesia Progressive leg weakness	13	C	6.0	37.7	6	C3/4 C4/5 C5/6	14
8	31	M	Neck pain arm dysaesthesia	10	D	7.4	36.6	6	C3/4	12
9	45	F	Neck pain arm dysaesthesia	5	D	8.2	35.5	7	C5/6	13
10	53	F	Neck pain	5	D	6.0	59.5	5	C5/6	12
11	36	M	Neck pain arm dysaesthesia Progressive leg weakness	6	D	5.6	32.0	5	C6/7	13
12	34	M	Neck pain arm dysaesthesia	6	D	7.4	54.5	6	C4/5	13
13	67	F	Neck pain arm and leg weakness	9	D	6.4	44.2	10	C4/5 C5/6	12
14	60	M	Neck pain arm and leg weakness	13	C	8.4	52.2	8	C3/4 C4/5	14
15	51	M	Neck pain arm dysaesthesia	8	D	8.6	49.4	8	C3/4 C4/5 C5/6	17
16	68	F	Neck pain leg dysaesthesia	12	D	6.2	23.2	5	C3/4	15
17	59	M	Neck pain arm dysaesthesia	12	D	9.7	29.1	9	C4/5 C5/6 C6/7	14
18	48	F	Neck pain arm dysaesthesia	15	C	7.7	47.9	3	C4/5 C5/6	13
19	33	M	Neck pain arm dysaesthesia Progressive leg weakness	15	C	8.4	49.1	7	C5/6	12
20	50	M	Neck pain arm dysaesthesia	13	C	6.7	62.1	5	C4/5 C5/6	16
21	47	M	Neck pain arm dysaesthesia	5	D	8.7	54.5	3	C4/5 C5/6	14
22	29	M	Neck pain Progressive numbness and weakness in the left arm and leg reduced temperature sensation on the right side of the body	8	D	5.9	31.2	8	C4/5 C5/6	13
23	67	M	Neck pain arm dysaesthesia	7	D	7.0	29.5	9	C3/4	14

**Fig. 1:**
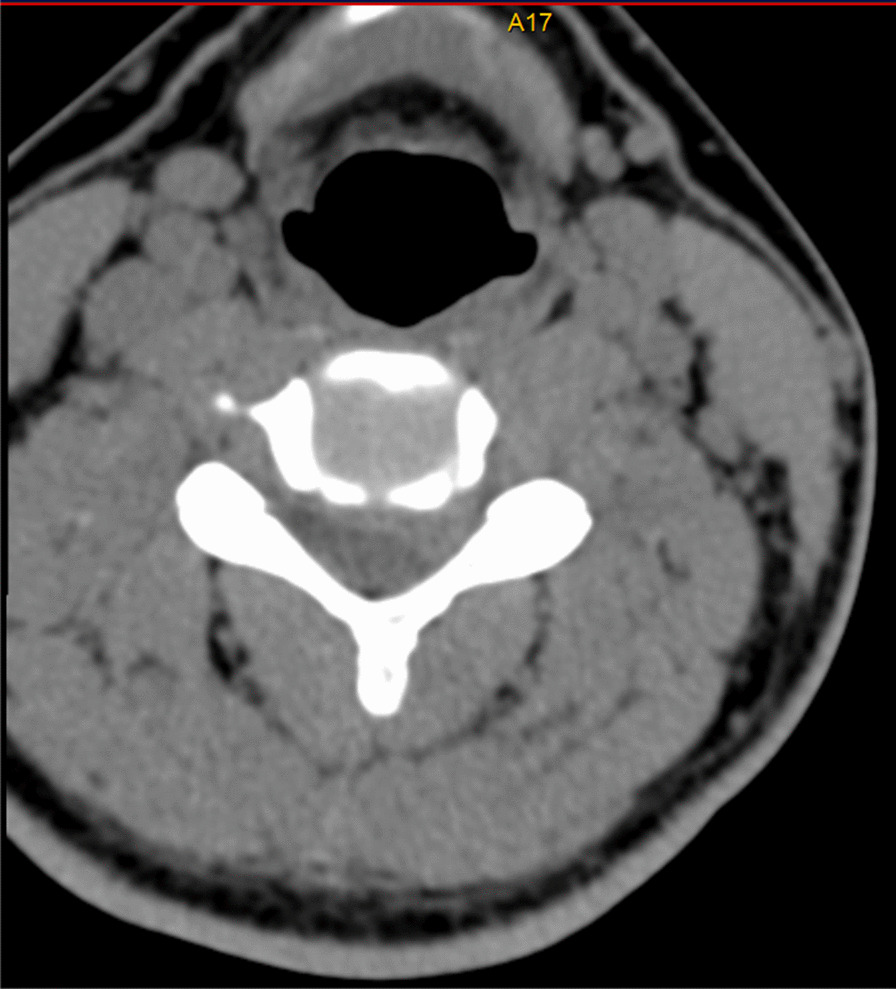
Preoperative CT showed giant cervical disc herniation

**Fig. 2:**
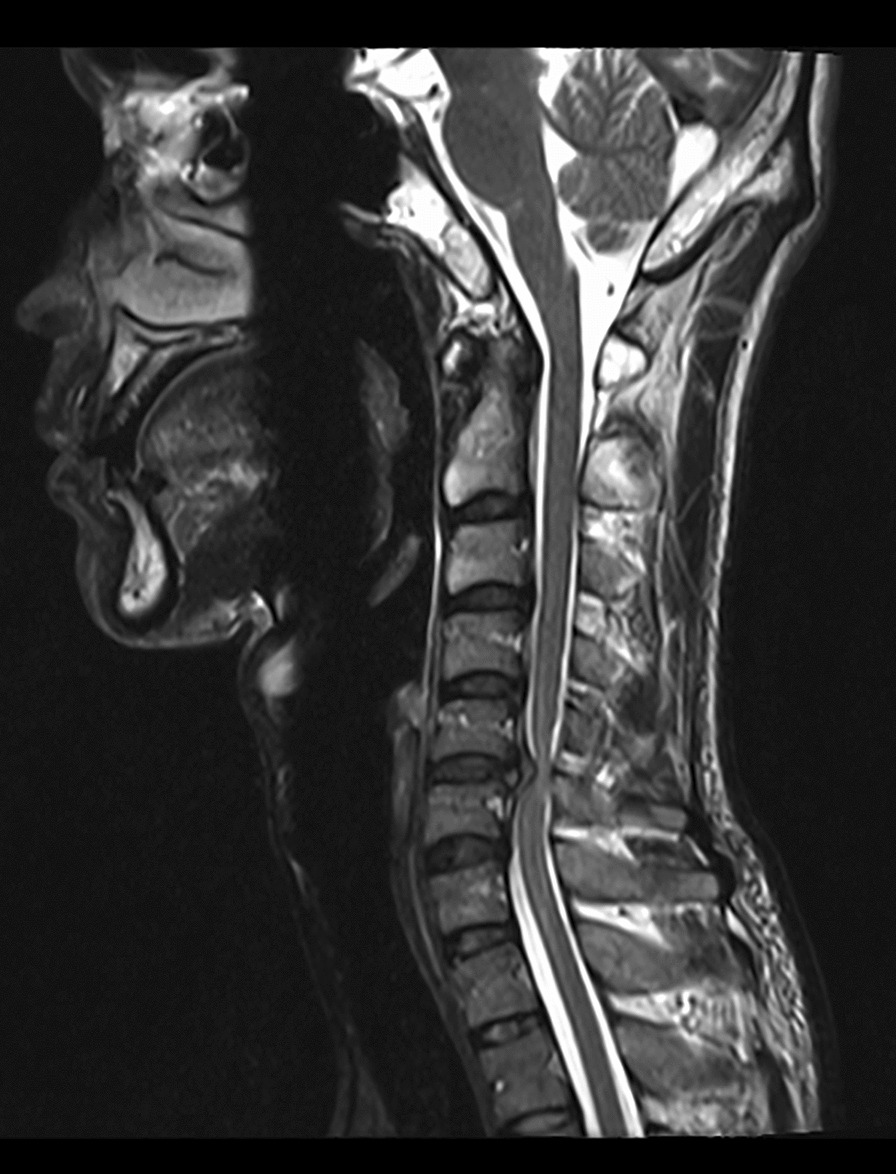
Preoperative MRI showed giant cervical disc herniation

**Fig. 3:**
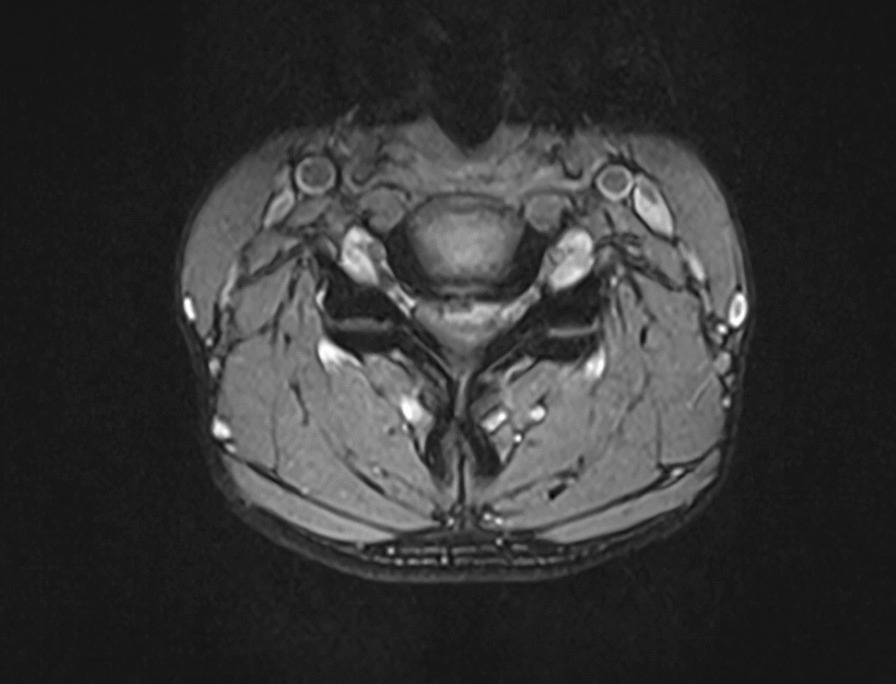
Preoperative MRI showed giant cervical disc herniation

All patients were examined by computed tomography (CT) (Fig. 1) and MRI before surgery to evaluate the degree of intervertebral disc invasion into the spinal canal (Figs. 2 and 3). Surgical records were reviewed in detail to determine the surgical time, intraoperative blood loss, and intraoperative complications. This study was carried out with the approval of the local hospital ethics committee.

### Surgical procedure

**Fig. 4:**
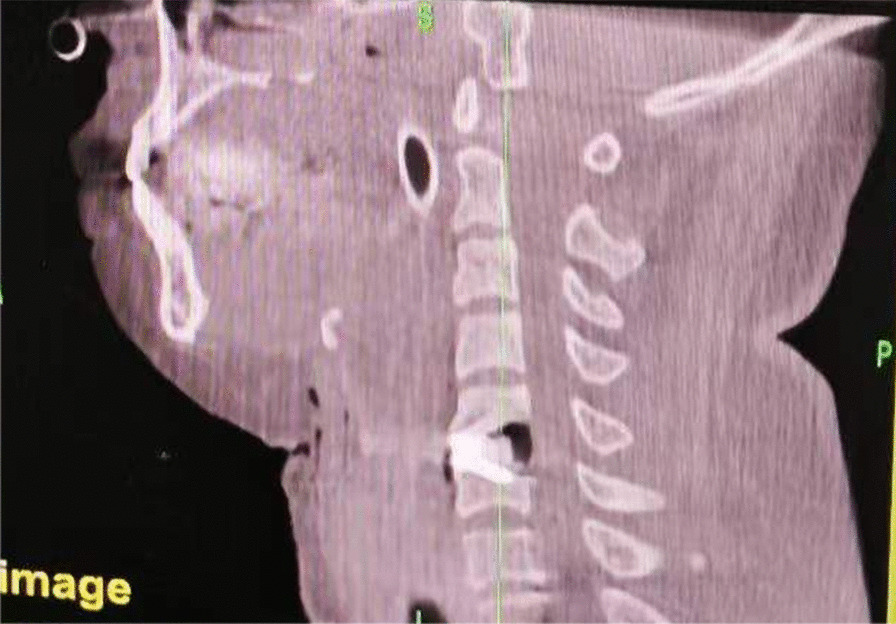
Sagittal reconstruction after the posterior edge of the vertebral body was subtly enlarged

**Fig. 5:**
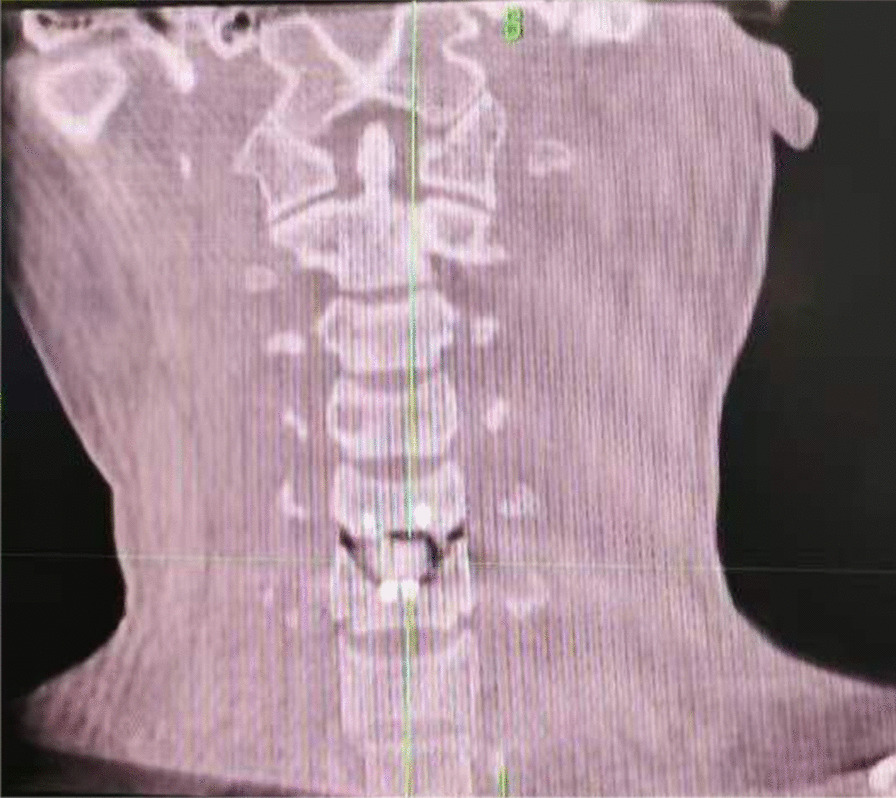
Coronal reconstruction after the posterior edge of the vertebral body was subtly enlarged

**Fig. 6:**
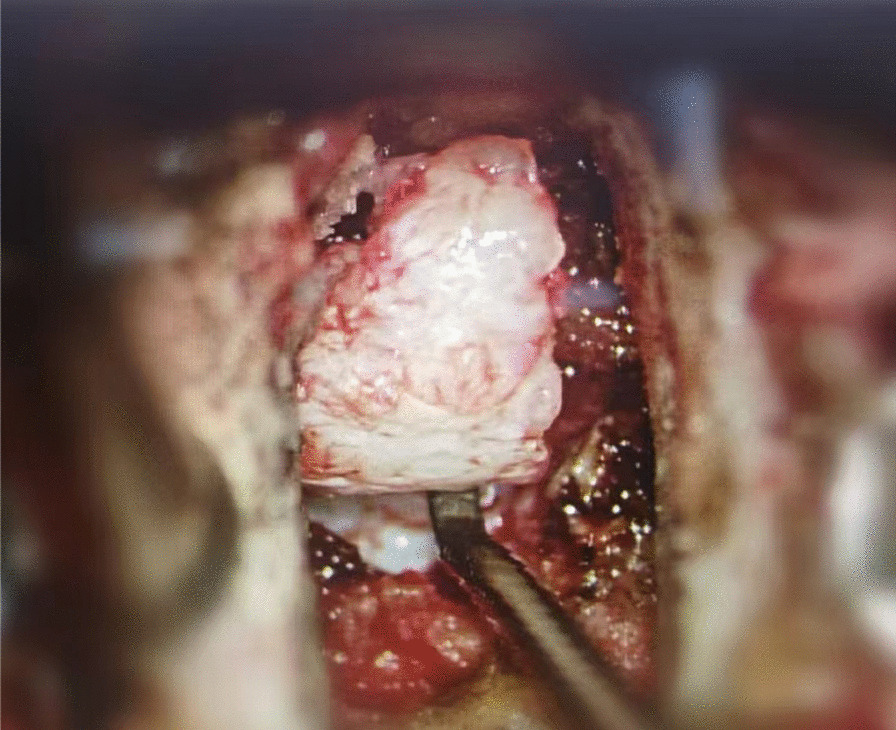
Intraoperative removal of cervical herniated disc

**Fig. 7:**
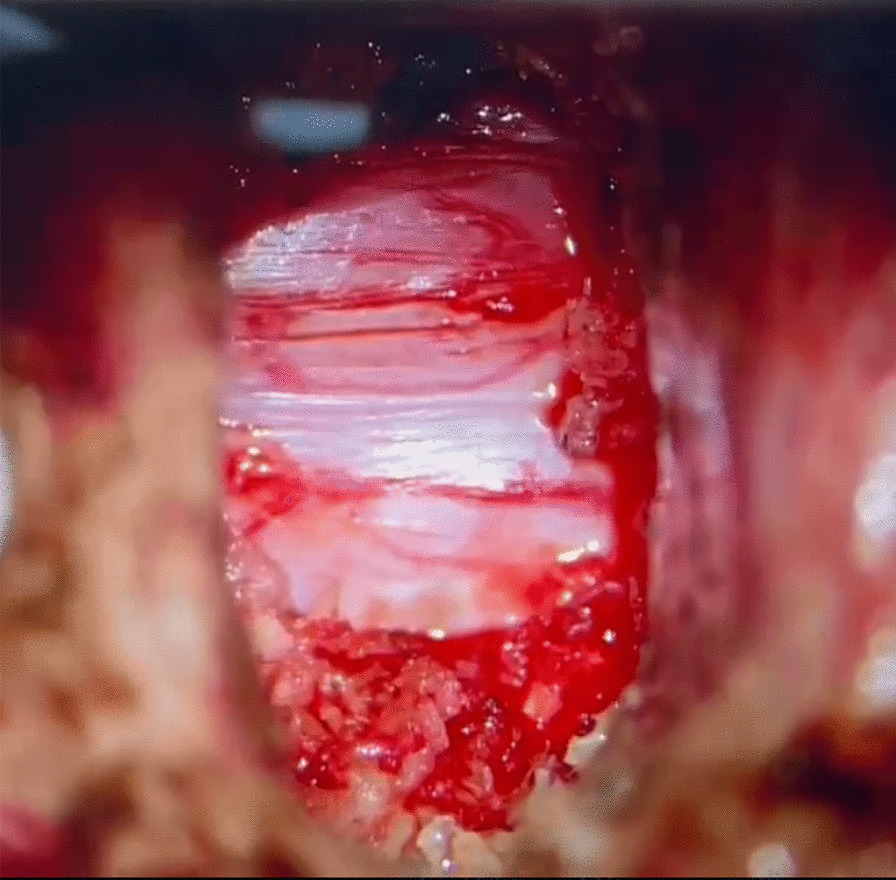
After decompression, the spinal cord was well pulsed and the probe was unobstructed

All surgeries were performed by the same group of surgeons. After successful anaesthesia, patients were placed in the supine position. Routine disinfection and towel laying were performed. After the prevertebral space was exposed and confirmed by the O-arm, the cervical intervertebral disc was scraped with a curette with the aid of a microscope, and the upper and lower soft endplates were treated. The osteophytes of the posterior edge of the vertebral body were removed with a grinding drill and ultrasonic bone knife, and the posterior edge of the vertebral body was subtly enlarged when necessary (Figs. 4, 5). After opening the posterior longitudinal ligament, the residual prominent nucleus pulposus was explored and removed. After decompression, the dural sac was reopened, the probe was clear, and the spinal cord was pulsating well (Figs. 6, 7). After washing and testing the model, the appropriate intervertebral notch fusion cage was implanted into the intervertebral space, confirmation of position by O-arm fluoroscopy, fixed with screws, and locked. The incision was washed repeatedly with normal saline and was checked to ensure no active bleeding. The drainage tube was retained, and the incision was sutured, layer by layer.

### Evaluation of efficacy and safety

In order to evaluate the efficacy of ACDF for the treatment of GCDH disease, visual analogue scale (VAS), Neck Disability Index (NDI), Japanese Orthopedic Association (JOA), and Frankel grade were analysed. The VAS, NDI, and JOA were measured and analysed at the pre-surgery outpatient appointment and three days, three months, and one year after surgery. The safety of ACDF was evaluated by examining the surgical complications, including vertebral artery injury, nerve injury, and postoperative infection. Postoperative infection assessment was based on serum leukocyte and inflammatory markers, including C-reactive protein and erythrocyte sedimentation rate.

### Data processing

SPSS 20.0 statistical software (IBM SPSS Statistics) was used for analysis. The data are expressed as mean ± standard deviation. Paired *t*-tests were used to evaluate changes in VAS scores, NDI scores, and JOA score before and after surgery (*P *< 0.05). All evaluations of curative effects were carried out by two doctors.

## Results

Between 2017 and 2021, 23 patients with GCDH were treated and neurological function monitoring was routinely performed during surgery. The clinical and imaging results showed that all 23 patients were able to tolerate the surgery, and all surgical times were very short. The average surgical time for single segments was 79.2 ± 24.9 min, and the average blood loss of single-segment surgery was 7.0 ± 5.7 ml.

**Fig. 8:**
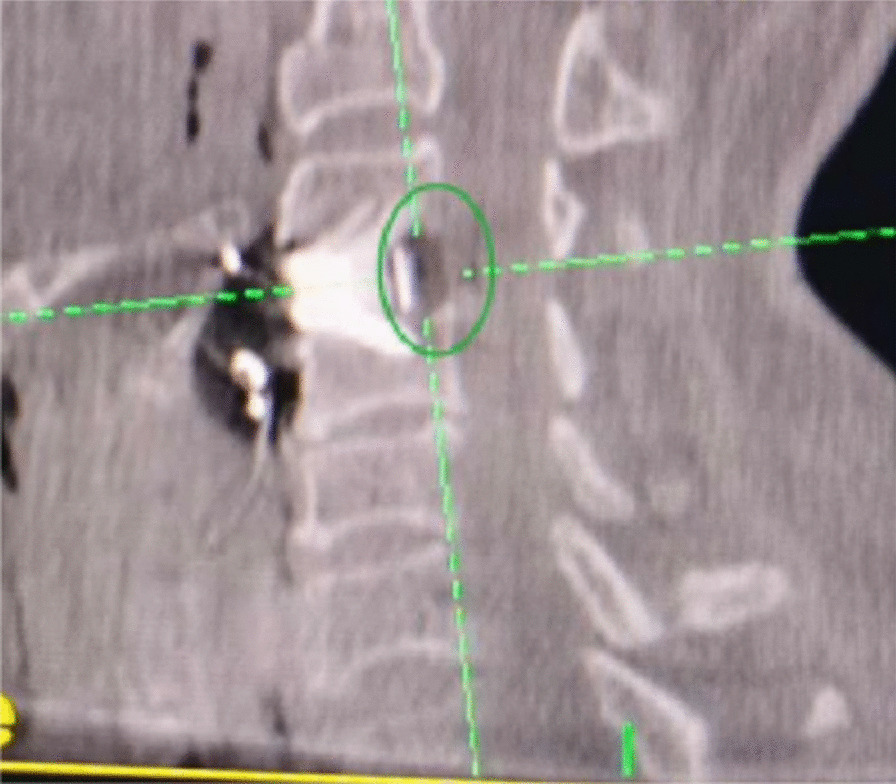
Postoperative CT showed that Complete bone debridement and adequate spinal cord decompression

**Fig. 9:**
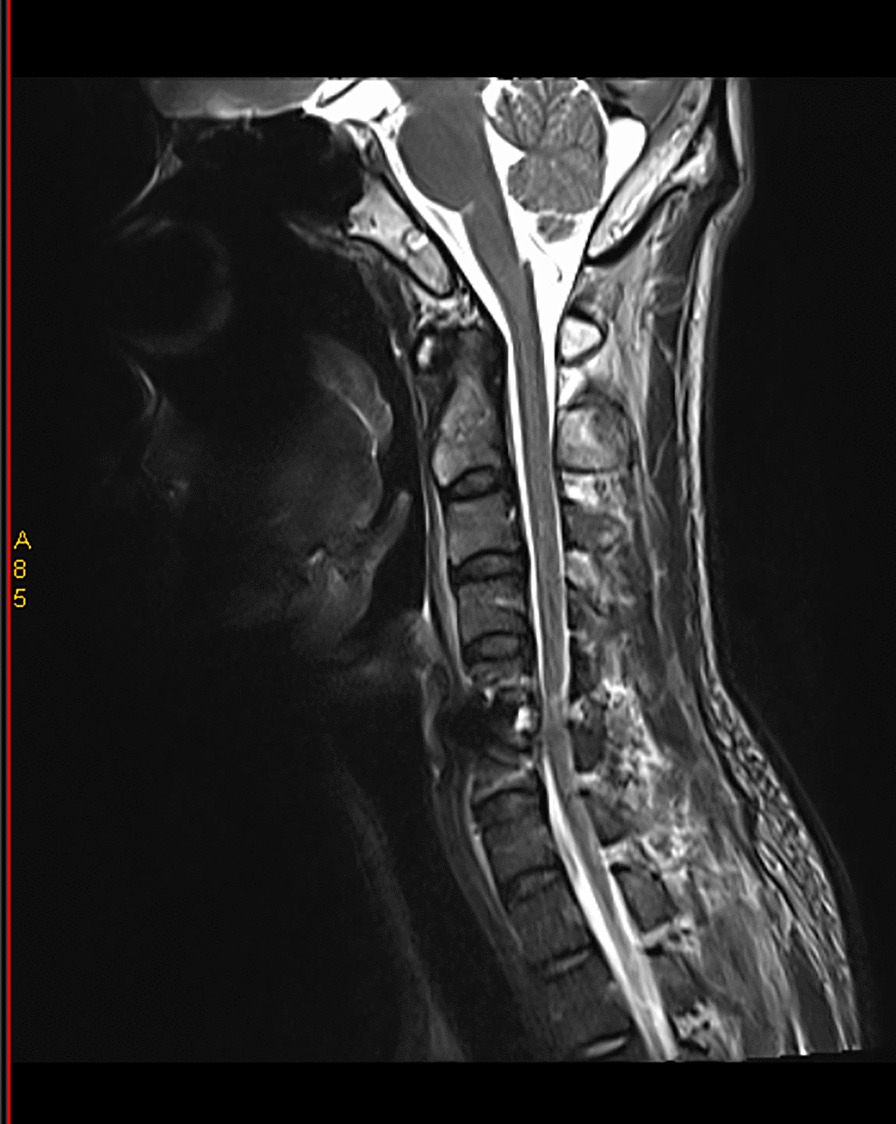
Postoperative MRI showed that giant cervical disc herniation had been removed

**Fig. 10:**
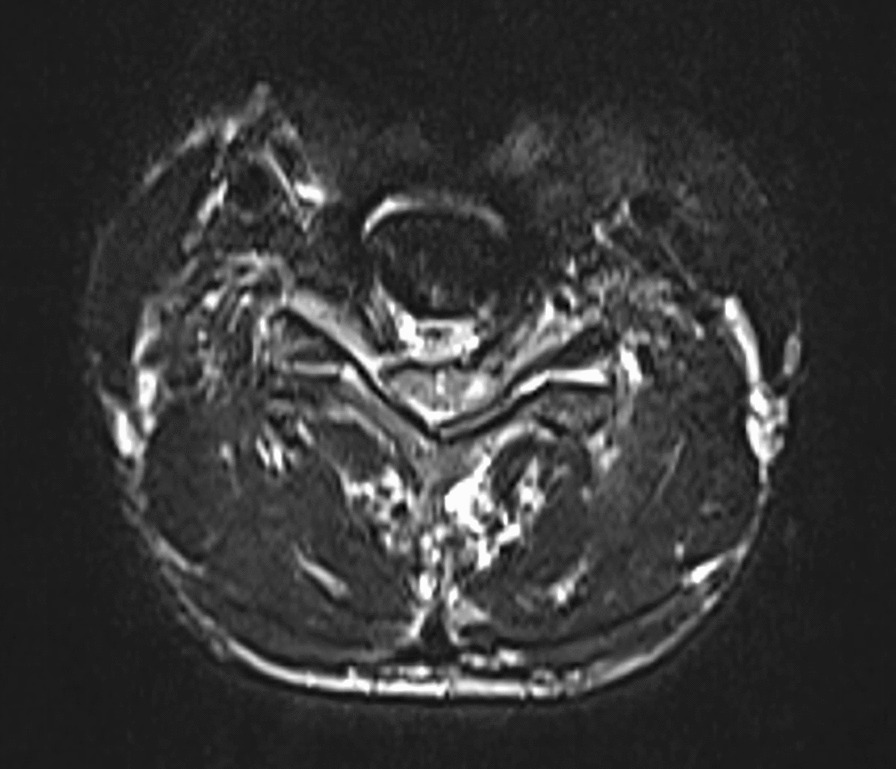
Postoperative MRI showed that giant cervical disc herniation had been removed

Spinal cord pulsation could be seen during the surgery. Postoperative CT (Fig. 8) and MRI scans (Figs. 9, 10) showed no residual intervertebral disc fragments in any cases, and all cases exhibited complete spinal cord decompression.

### Clinical outcomes

**Fig. 11:**
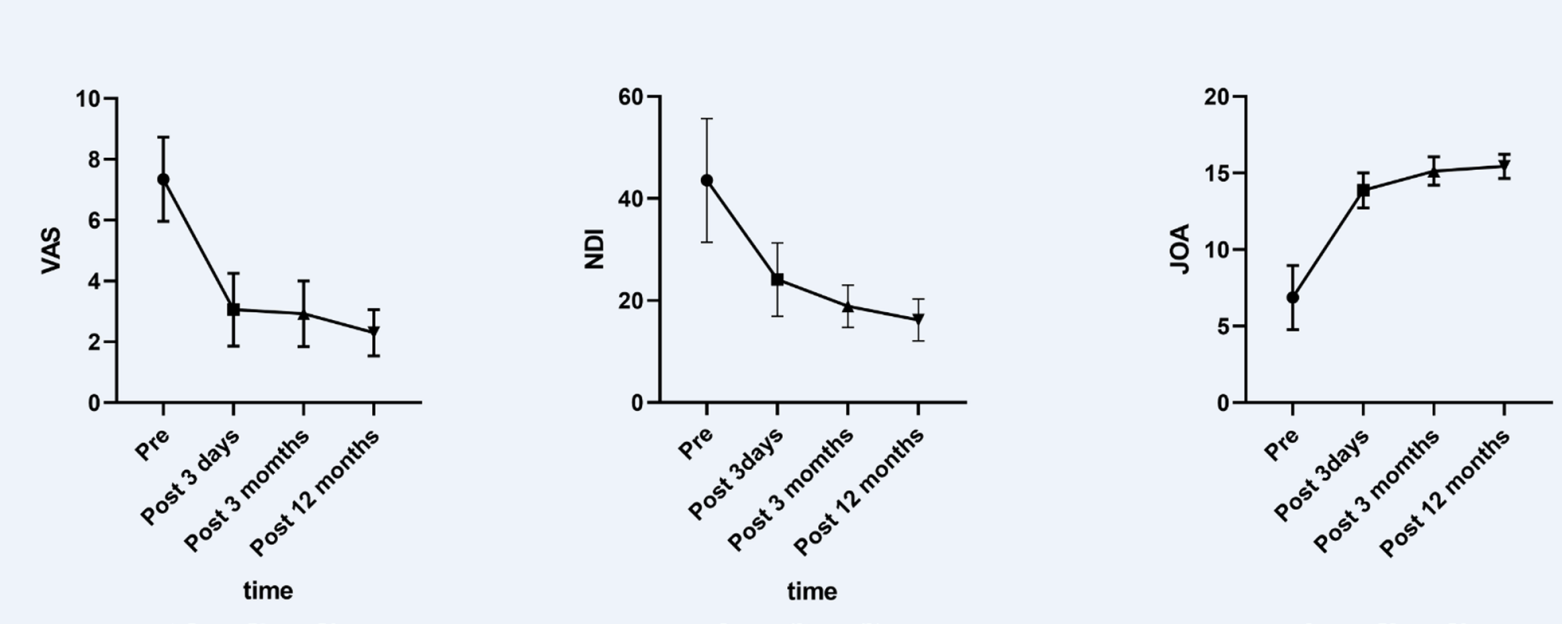
Changes in VAS scores, NDI scores, and JOA scores before and after surgery

Three days after surgery, all patients experienced significant reductions in neck pain and were able to walk and take care of themselves. The patients were followed up for 12–18 months (mean: 14 months). The average VAS score decreased from 7.3 ± 1.4 before surgery to 3.1 ± 1.2 three days after surgery (*P *< 0.001). The neck and shoulder pain of each patient was significantly improved. At the last follow-up, the average score remained at 2.3 ± 0.8, which was not significantly different compared with that immediately after surgery; thus, the pain relief effect of the surgery was long-lasting. There was a similar change in NDI scores. The average NDI score decreased from 43.6 ± 12.1 before surgery to 24.1 ± 7.2 three days after surgery and 16.2 ± 4.1 at the last follow-up. Thus, the patients’ dysfunction was significantly alleviated and their activities of daily living were significantly improved after surgery. The average JOA score increased from 6.9 ± 2.1 before surgery to 13.9 ± 1.1 three days after surgery (*P *< 0.001) and remained at 15.4 ± 0.8 at the last follow-up, which was not significantly different from that immediately after surgery (Fig. 11) (Table 2). The Frankel score of 17 patients increased by one grade (74%), the scores of three patients increased by two grades (13%), and the scores of three patients remained unchanged (13%). All patients achieved interbody fusion at the last follow-up.

**Table 2:** Efficacy evaluation

	Pre-ACDF	3 days after ACDF	3 months after ACDF	12 months after ACDF
VAS	7.3 ± 1.4	3.1 ± 1.2*	2.9 ± 1.1*	2.3 ± 0.8*
NDI (%)	43.6 ± 12.1	24.1 ± 7.2*	18.9 ± 4.2*	16.2 ± 4.1*
JOA	6.9 ± 2.1	13.9 ± 1.1*	15.1 ± 0.9*	15.4 ± 0.8*

*VAS* indicates visual analogue scale, *NDI* indicates Neck disability index, *JOA* indicates Japanese Orthopaedic Association scores

*P < 0.001 compared to preoperative value (Please see the Additional file 1 for the specific *P* value and CI)

### Complications

There were no complications, including nerve injury, vertebral artery injury, dural laceration, or oesophageal injury during the surgery. One patient developed transient hoarseness after the surgery, but the symptoms disappeared after a month of follow-up. No complications such as neurological deficits, cerebrospinal fluid leakage, wound infection, or pseudarthrosis were observed during the follow-up period, and no degeneration of adjacent segments was observed at the last follow-up.

## Discussion

GCDH is a rare type of cervical disc herniation. There is still controversy about the surgical method. Since most of the disc herniation is located in the front of the spinal cord, posterior surgery cannot directly remove the compressive material in front of the spinal cord, and the postoperative effect of the patient is poor. For GCDH, the anterior approach is still the best approach, and the commonly used anterior surgery methods are ACCF and ACDF. ACCF removes part of the vertebrae and intervertebral discs to decompress the spinal cord and nerves. Although ACCF has the advantage of directly removing the nucleus pulposus and other compressive substances, ACCF has many complications, including vertebral artery, dural tear, cerebrospinal fluid leakage, graft displacement and other complications. Moreover, patients after ACCF have been in bed for a long time and cannot recover as soon as possible. ACDF removes the intervertebral disc through a less invasive approach to the intervertebral space and relieves the compression of the intervertebral disc on the spinal cord. Compared with ACCF, it has the advantages of less blood loss, shorter hospital stay and fewer postoperative complications. However, the ACDF intervertebral space is limited, and the anatomical structure of the surgical area is difficult to clearly identify under traditional direct vision. There is a risk of damaging the dura mater, nerve roots, and even vertebral arteries during the removal of huge cervical disc herniation. The use of a microscope can improve the clarity of the visual field, and its magnification can help to identify the tiny blood vessels and nerves and reduce their damage, at the same time, it can improve the complete removal of the lesions and improve the surgical effect. Ultrasonic bone dissector has good tissue selectivity and hemostatic properties and has been safely used to promote osteotomies in various spinal operations to promote the surgical process without damaging the nerves. The combined use of microscope and ultrasonic bone dissector makes it possible for ACDF to treat huge cervical disc herniation. As far as we know, there are few studies on the treatment of huge cervical disc herniation with ACDF. The purpose of this study is to evaluate the feasibility and efficacy of ACDF in the treatment of GCDH patients with the aid of microscopes and ultrasonic bone dissector.

This study included 23 patients with GCDH (including three patients with disc herniation with calcification), all of whom underwent ACDF surgery and were followed up for more than 12 months. All patients showed significant improvement in VAS score, NDI score, and JOA score at 3 days after surgery compared with those before surgery, and the difference was statistically significant, indicating that ACDF achieved satisfactory results in treating GCDH, effectively relieving neck and shoulder pain and improving nerve function. Moreover, its effects were maintained until the last follow-up evaluation. In patients with giant cervical disc herniation, the anterior approach is still the best approach because it is the only way to perform true anterior decompression of the spinal cord, since most of the disc herniation is located anterior to the spinal cord and the posterior approach does not directly remove the anterior spinal cord compressor. Williams et al. [5] Direct removal of herniated or free disc nucleus pulposus, bone fragments, thickened or posterior longitudinal ligaments through an anterior cervical subtotal laminectomy results in thorough, adequate, direct and effective decompression. However, some scholars [10] suggested that due to the lack of reliable stability of the bone graft interface, delayed healing or even non-healing of the bone graft may occur, and the bone graft may be dislodged in severe cases. In this study, all patients achieved good neurological recovery and significant improvement in neck pain symptoms, and no complications such as neurological deficits or pseudoarthrosis were found during the follow-up period, and no degeneration of adjacent segments was detected at the last follow-up, so it can be considered that ACDF is effective in treating GCDH.

Liu et al. [11] suggested that during ACDF for GCDH surgery, the patient's spinal canal reserve gap is extremely narrow and the spinal cord is severely compressed, and coupled with the influence of surgical instruments, a slight inadvertence may easily damage the spinal cord and aggravate neurological symptoms, or even cause respiratory and cardiac arrest. Lin et al. [12] by first decompressing the spinal canal posteriorly, the reserve space of the spinal canal can be fully expanded to increase the cushion space of the spinal cord, reduce the stimulation of the spinal cord by anterior surgery, and reduce the risk of spinal cord injury, and then perform anterior decompression and internal fixation with bone grafting and fusion to avoid the limitation that simple anterior or posterior repositioning and fixation cannot simultaneously rebuild the stability of the anterior column and posterior structures. However, the operation is more invasive, the operation time is significantly longer, the bleeding is higher, and the physical condition of the patient is more demanding; the position is changed several times during the operation, which may cause spinal cord injury if not properly protected.

In the 23 patients in this study, there were no intraoperative spinal cord injuries, vertebral artery injuries, or nerve injuries. Use the microscope during each surgical procedure, providing a good field of view and reducing the risk of arterial and nerve injury, so microscope-assisted ACDF for GCDH improves the safety of the procedure. Meanwhile, Microscope-assisted combination of ultrasonic bone dissector to treat huge cervical disc herniation has achieved good clinical results. The possible reasons are: 1. Microscope-assisted ACDF can magnify and clarify the narrow surgical field and clearly show the capillary and nerves. It allows the surgeon to find the remaining nucleus pulposus that is difficult to detect under the naked eye, making the decompression more thorough. 2. The combination of high-speed grinding brick and ultrasonic bone dissector were used in ACDF. Then, used the ultrasonic bone dissector to cut the dura and the bone adjacent to the nerve root for a deeper and more precise cut. This combined method is safer and more efficient than simply using one of them. In the current study, surgeries were performed with the aid of a microscope. The use of a microscope allowed for a bright, clear, and magnified field of vision during surgery. A high-speed grinding drill and ultrasonic bone knife were used to remove the osteophytes on the posterior edge of the vertebral body and enlarge the posterior edge of the vertebral body. Usually, 1/5 of the posterior edge of the vertebral body was removed in order to expand the operating space. This allowed clear vision of the posterior longitudinal ligament. The posterior longitudinal ligament is divided into a deep and shallow layer. The shallow layer is often broken due to disc herniation. Some disc fragments are in the deep and shallow layers of the posterior longitudinal ligament, so the free nucleus pulposus should be explored and removed. With ACDF, the deep layer of the posterior longitudinal ligament is usually removed, because (1) this can prevent omission of disc fragments, and (2) the posterior longitudinal ligament thickens and has high tension, which may still oppress the spinal cord. The high tension in the posterior longitudinal ligament may be due to the long-term compression produced by the huge cervical disc herniation. In short, all surgeries were performed with the assistance of a microscope and all surgeries achieved decompression of the spinal cord and nerve with good results.

Furthermore, in this study, the Frankel grade of 17 patients increased by one grade (74%), the grades of 3 patients increased by two grades (13%), and the grades of 3 patients did not change (13%). Despite no change in Frankel grade, these patients were satisfied with the results of the surgery because their neurological function did not deteriorate further and their pain was relieved as compared to before the surgery. In this group of cases, the symptom duration of patients without neurological improvement was longer than that of the other patients, and the average conservative treatment time was more than six months. Although the sample size was small, there was no statistically significant effect of conservative treatment duration on postoperative neurological function recovery, which still suggests that GCDH may require early surgical treatment.

In addition, among the included cases found in this study, C3–C4 disc herniation is the most common. But previous studies [13] have reported that cervical spondylotic myelopathy is most commonly observed in C5–C6, followed by C6–C7, C4–C5, and C3–C4. This may be because the C3–C4 spinal canal space is relatively large, and thus, the symptoms of patients with mild disc herniation are not obvious. When giant disc herniation compresses the spinal cord, patients will only come to see a doctor when they have obvious medullary symptoms.

This study has several limitations. Firstly, this study included only a small number of cases, the follow-up time was short, and there were no statistics on the long-term complications of the patients. Second, since this is only a preliminary study, a case–control design comparing the efficacy of ACDF with other procedures was not employed. Finally, the economy of the surgery was not considered. Therefore, future studies of patients with GCDH need to be designed more carefully to include more patients and longer follow-up times in order to verify the efficacy of this surgery.

## Conclusion

This study shows that microscopic ACDF treatment of GCDH is feasible and can be used to safely remove herniated disc fragments, effectively relieve spinal cord compression, and improve neurological status. The surgical effects are maintained for a long period of time.

### Supplementary Information

**Additional file 1.** VAS, NDI, JOA specific P value and CI detailed information.

## Data Availability

Anyone who wishes to access those data could contact the corresponding author (Guangye Wang) by email.
