# Supplementary material for: Anterior cervical discectomy and fusion for the treatment of giant cervical disc herniation
Source: J Orthop Surg Res. 2023 Sep 13;18:683. doi: 10.1186/s13018-023-04036-5 (PMC10500809; doi:10.1186/s13018-023-04036-5)
Supplement: Supplementary file 1 — Additional file 1. VAS, NDI, JOA specific P value and CI detailed information. [file 13018_2023_4036_MOESM1_ESM.docx]

VAS、NDI、JOA specific P value and CI detailed information

VAS

| **Paired Samples Test** | | | | | | | | | |
| --- | --- | --- | --- | --- | --- | --- | --- | --- | --- |
|  | | Paired Differences | | | | | t | df | Sig. (2-tailed) |
|  |  | Mean | Std. Deviation | Std. Error Mean | 95% Confidence Interval of the Difference | |  |  |  |
|  |  |  |  |  | Lower | Upper |  |  |  |
| Pair 1 | Pre-ACDF - 3 days after ACDF | 4.28261 | 1.78673 | .37256 | 3.50997 | 5.05525 | 11.495 | 22 | .000 |
| Pair 2 | Pre-ACDF - 3 months after ACDF | 4.41739 | 1.79814 | .37494 | 3.63982 | 5.19497 | 11.782 | 22 | .000 |
| Pair 3 | Pre-ACDF - 12 momths after ACDF | 5.04348 | 1.57965 | .32938 | 4.36039 | 5.72657 | 15.312 | 22 | .000 |

NDI

| **Paired Samples Test** | | | | | | | | | |
| --- | --- | --- | --- | --- | --- | --- | --- | --- | --- |
|  | | Paired Differences | | | | | t | df | Sig. (2-tailed) |
|  |  | Mean | Std. Deviation | Std. Error Mean | 95% Confidence Interval of the Difference | |  |  |  |
|  |  |  |  |  | Lower | Upper |  |  |  |
| Pair 1 | Pre-ACDF - 3 days after ACDF | 19.45652 | 12.70064 | 2.64827 | 13.96435 | 24.94869 | 7.347 | 22 | .000 |
| Pair 2 | Pre-ACDF - 3 months after ACDF | 24.70000 | 12.19426 | 2.54268 | 19.42681 | 29.97319 | 9.714 | 22 | .000 |
| Pair 3 | Pre-ACDF - 12 momths after ACDF | 27.37826 | 11.54651 | 2.40761 | 22.38518 | 32.37134 | 11.372 | 22 | .000 |

JOA

| **Paired Samples Test** | | | | | | | | | |
| --- | --- | --- | --- | --- | --- | --- | --- | --- | --- |
|  | | Paired Differences | | | | | t | df | Sig. (2-tailed) |
|  |  | Mean | Std. Deviation | Std. Error Mean | 95% Confidence Interval of the Difference | |  |  |  |
|  |  |  |  |  | Lower | Upper |  |  |  |
| Pair 1 | Pre-ACDF - 3 days after ACDF | -7.00000 | 2.11058 | .44009 | -7.91268 | -6.08732 | -15.906 | 22 | .000 |
| Pair 2 | Pre-ACDF - 3 months after ACDF | -8.26087 | 2.41618 | .50381 | -9.30571 | -7.21603 | -16.397 | 22 | .000 |
| Pair 3 | Pre-ACDF - 12 momths after ACDF | -8.56522 | 2.50138 | .52157 | -9.64690 | -7.48354 | -16.422 | 22 | .000 |
